# The biology and in vitro propagation of the ornamental aquatic plant, *Aponogeton ulvaceus*

**DOI:** 10.1186/s40064-016-3041-4

**Published:** 2016-09-26

**Authors:** Melissa Yit Yee Kam, Li Chin Chai, Chiew Foan Chin

**Affiliations:** Faculty of Science, School of Biosciences, The University of Nottingham Malaysia Campus, Selangor Darul Ehsan, Malaysia

**Keywords:** *Aponogeton ulvaceus*, Circumnutation, Glandular trichomes, Callus induction, Plant growth regulators, Immature tuber

## Abstract

*Aponogeton ulvaceus* Baker (Aponogetonaceae) is a commercially important ornamental aquatic plant species with traditional medicinal uses. Due to the low survival rate of seedlings, propagation by conventional means has been met with many difficulties. In this study, botanical aspects of *A. ulvaceus* were examined with regards to the morphology, anatomy and physiology of the plant and an efficient protocol for its in vitro propagation using immature tuber explants has been established. The existence of glandular trichomes on the leaves was discovered and the occurrence of circumnutation in *A. ulvaceus* has been demonstrated. Immature tuber segments with meristems were cultured on MS medium supplemented with various combinations (0, 1, 2, and 3 mg/L) of BAP and NAA for callus induction. The highest percentage of callus production (100 %) was obtained in two different treatments: 1 mg/L BAP and 3 mg/L NAA, and 2 mg/L BAP and 3 mg/L NAA. For shoot and root organogenesis, the combination of 1 mg/L BAP and 1 mg/L NAA was shown to be significant for *A. ulvaceus* regeneration when compared to control, which yields a mean shoot and root number of 22.50 and 29.50 respectively. The current protocol is the first reported successful establishment of in vitro clonal propagation of *A. ulvaceus*.

**Electronic supplementary material:**

The online version of this article (doi:10.1186/s40064-016-3041-4) contains supplementary material, which is available to authorized users.

## Background

**Fig. 1:**
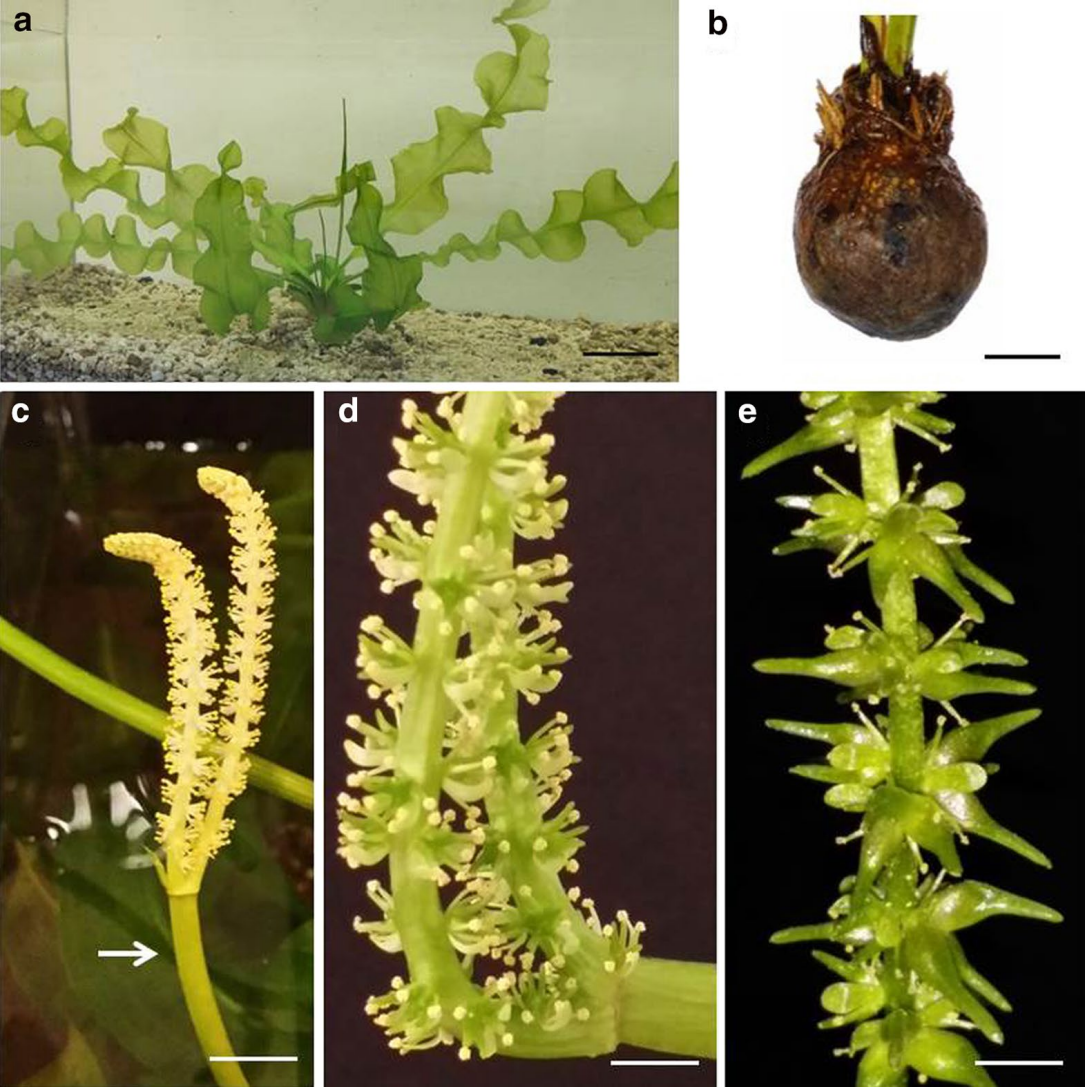
Morphology of *A. ulvaceus*. **a** Aquarium grown *A. ulvaceus* plant; *scale bar* 5 cm. **b** A mature tuber with shoots arising from the shoot apical meristem; *scale bar* 1 cm. **c** An immature inflorescence borne on rachis (*arrow*); *scale bar* 1 cm. **d** Individual flowers along spikes of mature inflorescence; *scale bar* 0.5 cm. **e** Ripe follicles of *A. ulvaceus*; *scale bar* 0.5 cm. Note the distinct, curved beak and shrivelled stamens

*Aponogeton ulvaceus* Baker (Madagascar water lettuce), is a submerged aquatic monocot native to the rivers of central and northern Madagascar (James 1986; Les et al. 2005; Azan 2011). This herbaceous perennial belongs to the family Aponogetonaceae (order Alismatales). Along with other 51 *Aponogeton* species of perennial aquatics, this plant predominantly distributes in the subtropical and tropical areas of Africa, Madagascar, India, Sri Lanka, Southeast Asia, Australia and New Guinea (Robinson 2011; Grímsson et al. 2014). It thrives well in both stagnant and flowing waters with varying light conditions (extreme shade to intense sunlight) and fluctuating water temperature (22–28 °C) (James 1986). The leaves, arranged in a rosette, may reach up to 60 cm in length and 10 cm in width (Fig. 1a) (Thabrew 2014). They are borne on petioles of equal length arising from a slightly hairy, cone-shaped tuber (Fig. 1b). This Madagascan species survives seasonal dry periods by entering dormancy, whereby the dormant tuber functions as food storage (Ingrouille and Eddie 2006). Inflorescences with a yellow colour grow on long peduncles of varying length according to the water depth (Fig. 1c). The self-sterile inflorescences are composed of 2 spikes and may attain a maximum length of 15 cm (Azan 2011). Flowers are hypogynous, small and sessile (Fig. 1d). They are arranged more or less spirally along the rachis. The ripe fruit of *A. ulvaceus* is a follicle and it is schizocarpic with a mostly distinct terminal, often curved beak (Fig. 1e) (Lye 1989; Grímsson et al. 2014).

The genus *Aponogeton* has been regarded as ideal for use as aquarium plants or water garden ornamentals (Les et al. 2005; Azan 2011). It comprises some of the commercially important aquatic species utilized in the aquatic plant trade. Among the species in the family of Aponogetonaceae, *A. ulvaceus* has stood out as the popular ornamental aquarium plant that is large and imposing. In addition to its commercial importance and aesthetic appeal, *Aponogeton* is reported to have medicinal properties such as antidiabetic activity (Munasinghe et al. 2010; Dash et al. 2014). It is also used for treating stomach disorder as well as reviving the digestive system (Shankar and Mishra 2012). Likewise, *Aponogeton* forms part of the diet of the Anuradhapura District of Sri Lanka (Munasinghe et al. 2010) and some regions of Africa (Pemberton 2000) whereby the consumption of flowers, flower stalks and tubers has been reported. In Cape South Africa, for instance, inflorescence of *A. distachyos* (waterblommetjie) is a domesticated food crop and a unique South African delicacy.

The unique contortion of the lamina under high light irradiance, resulting in the formation of twisted leaves in a cork-screw manner gave *A. ulvaceus* its intricate morphology (Czaja 1930). It appears that there are some form of associations between such behaviour and light-induced stress. This could possibly be a strategy of *A. ulvaceus* to survive excess illumination through leaf movement by orientating and exposing the abaxial surface to light instead of the adaxial side. Intriguingly, the leaves may develop a rufescent colouration under the same conditions (James 1986; Thabrew 2014).

Due to the low survival rate of seedlings in the early stages and thus, difficult to propagate conventionally (Sweeney 2008), *A. ulvaceus* is in short supply. To meet the high demands for *A. ulvaceus*, most plants offered for sale in the aquarium trade are harvested from native populations and overharvesting exert pressures on wild sources, resulting in diminutions in density and abundance.

In vitro culture is an excellent alternative method for the large-scale production of this valuable Malagasy species, mitigating both supply and quality predicaments. To date, numerous experimental studies on aquatic plant tissue culture have been reported. Aquatic plant species that have been successfully propagated in vitro include *A. madacasgariensis* (Carter and Gunawardena 2011), *Cryptocoryne wendtii* (Stanly et al. 2011), *Hygrophila polysperma* (Roxb.) T. Anderson (Çinar et al. 2013), *Myriophyllum spicatum* L. (Zhou et al. 2006), *Nymphoides indica* (Jenks et al. 2000), *Pogostemon helferi* (Wangwibulkit and Vajrodaya 2016) and *Potamogeton crispus* L. (Zhou et al. 2006). To the best of our knowledge, this is the first report describing the micropropagation of *A. ulvaceus*. In this study, specific aspects of *A. ulvaceus* morphology, anatomy and physiology were investigated in order to supplement the earlier literature and an efficient clonal micropropagation system via indirect organogenesis was established.

## Methods

### Plant materials and culture conditions

*A. ulvaceus* plants were supplied by local aquarium plant trader. The plants were maintained in water-filled tanks until flowering. Mature flowers were cross-pollinated with fresh pollens from plants of the same species. Spikes with seed-bearing follicles were disinfected in 70 % (v/v) ethanol for 30 s, followed by 0.4 % (v/v) sodium hypochlorite for 20 min. Seeds were subsequently removed from follicles and germinated on basal MS medium (Murashige and Skoog 1962) (Duchefa Biochemie B. V.) containing 3 % (w/v) sucrose (Fisher Scientific) and 0.35 % (w/v) Phytagel (Sigma-Aldrich). To prevent desiccation, seeds were submerged in liquid MS medium. All media were adjusted to pH 5.8 and autoclaved at 121 °C for 15 min. Cultures were maintained at 25 ± 1 °C under a 16 h photoperiod (40 μmol m^−2^ s^−1^) provided by cool-white fluorescent lamps (Akari TLD36W/54). In vitro-raised plantlets were subcultured at 21-day intervals into fresh MS basal medium.

### Morphological, anatomical and physiological studies

Developing seedling and mature plant samples were used for morphological analysis. Plant samples were subjected to imaging with an AZ100 dissecting microscope equipped with a Digital Sight DS-5Mc camera (Nikon) to characterize the distinct developmental features of *A. ulvaceus*. To identify the anatomical structures, transverse sections of fresh leaf midrib and petiole samples were prepared and mounted in sterile distilled water to prevent desiccation for microscopic examination.

An interesting phenomena with respect to the physiology of *A. ulvaceus* known as circumnutation was investigated. Plant movements are often considered as a result of an environmental stimulus reaction. Circumnutations, however, are autonomous helical movements of plant organs whereby the tips outline a circular, full-ellipsis, pendulum-like shape or irregular zigzags over a sufficiently long period (using time-lapse video method) (Stolarz 2009). For circumnutation observations and measurements, a mature plant was cultivated in a shallow-water acrylic tank measuring 26 cm in depth from the water surface to the base of the plant at room temperature (25 ± 1 °C) under continuous illumination. Time-lapse video of the oscillating growth patterns of a 4-day-old shoot apex was recorded by lapse it (interactive universe). Camera parameters remained constant throughout the experiment and time-lapse images were recorded one frame per 5 min intervals for 9 h. The plant was filmed from the top-view. Time-lapse images were digitized using *Circumnutation Tracker* (*CT*) (Stolarz et al. 2014) and Microsoft Excel programs.

### Callus induction and organogenesis

Immature tubers with meristems obtained from in vitro-raised plantlets were used as explant materials. Tuber explants were longitudinally dissected into four equal sections and cultured on MS medium supplemented with different concentrations of BAP (0, 1, 2, and 3 mg/L) in combination with NAA (0, 1, 2 and 3 mg/L) for callus induction and subsequent regeneration (Carter and Gunawardena 2011). To prevent desiccation, liquid MS was poured over the explants to submerge the entire tuber segment. Plant growth regulators (PGRs) were filter-sterilized (0.2 μm pore size, Minisart, Sartorius Stedim Biotech) before being added to molten autoclaved MS. The experimental design was completely randomized with four culture samples per treatment and each treatment was replicated thrice under the same conditions as above. The frequency of explants producing callus was determined after 28 days of culture. Subsequently, the evaluation was repeated 56 days later on the shooting and rooting responses. Likewise, the number of shoots and roots per explant was determined.

### Statistical analysis

Statistical tests were performed with Genstat 10 for Windows. Percentage data of initial callusing was analysed using Kruskal–Wallis test. Prior to statistical analysis, the mean number of both shoots and roots at day 28 was subjected to logarithm transformation. The square root was used for the mean number of shoots and roots analysis at day 56 and arcsine transformation for the percentage of organogenic explants experiment in day 28 and 56. Data were tested for normality and analysed using one-way ANOVA. The mean data were compared using Duncan’s multiple range test with a 0.05 confidence interval.

## Results

### Morphological features

**Fig. 2:**
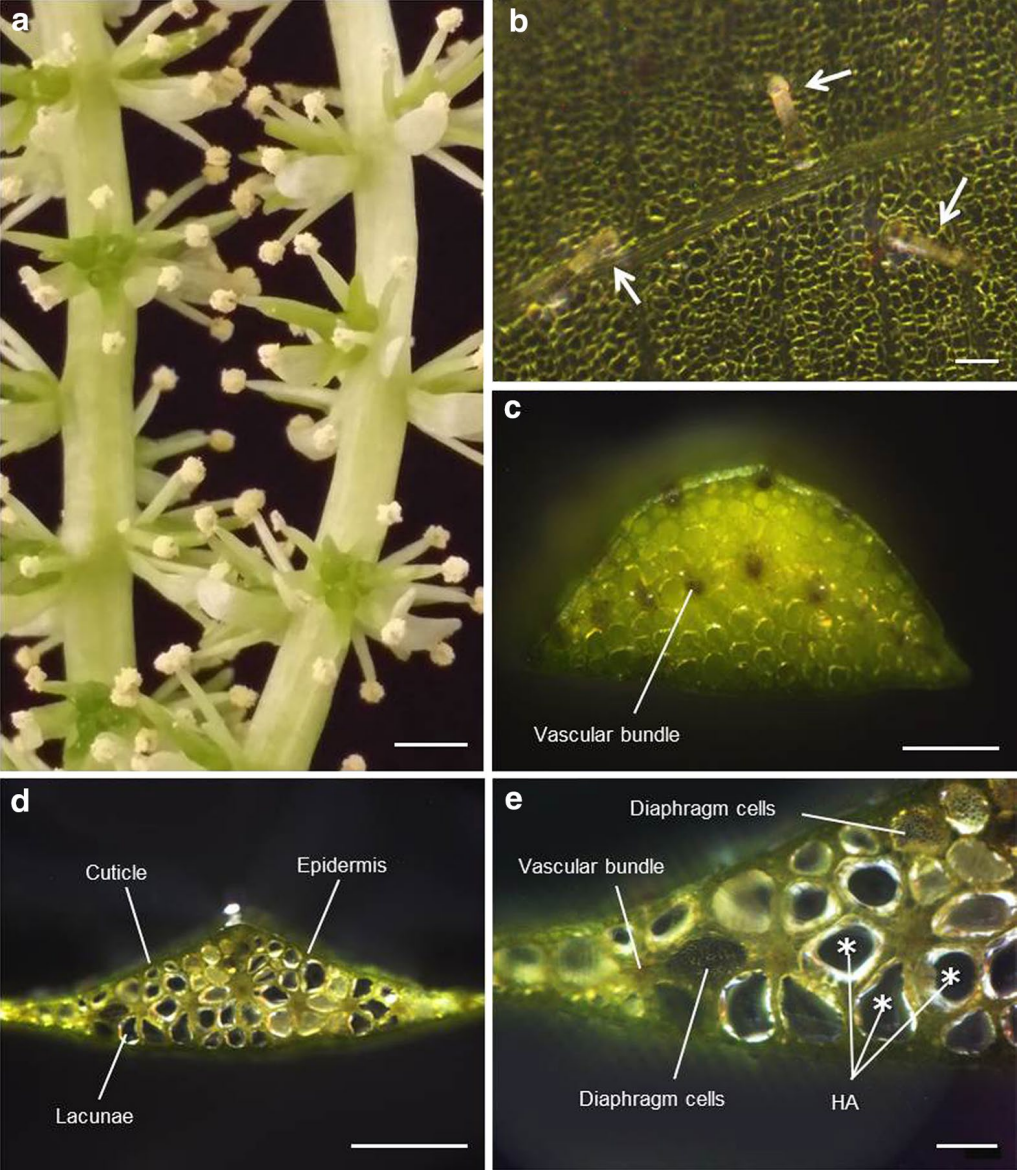
Morphological and anatomical features of *A. ulvaceus*. **a** Close up image of individual flowers of *A. ulvaceus*; *scale bar* 0.2 cm. **b** Glandular trichomes (*arrows*) on leaf surface; *scale bar* 100 μm. **c** Cross section of a petiole; *scale bar* 500 μm. **d** A transverse section of the leaf midrib; *scale bar* 1000 μm. **e** A closer view of the honeycomb aerenchyma (HA), extensive lacunae and diaphragms; *scale bar* 200 μm

Owing to the lack of literature on this economically important aquatic macrophyte, little is known about its morphology and almost nothing in regards to the anatomical structures of *A. ulvaceus*. Therefore, the present study examined several characteristics of this plant species and provided a detailed description on these aspects. Observational data showed that *A. ulvaceus* is monoecious, with perfect flowers, where both male and female organs occur together (Fig. 2a). The stamens generally appear in 4–7, but most often six surrounding the female structures in the centre. Anthers are of 2-thecate and pollens are always yellow. Likewise, the darker green carpels exist in 3–5 (usually three free carpels), unilocular and are pear-shaped. Each carpel will give rise to individual fruit. The isobilateral bright green (sometimes pale green) leaves are translucent and wavy; blades are often undulated and sometimes contorted (Fig. 1a). The blades usually have a distinct midrib with three or four pairs of parallel, confluent nerves connected by numerous cross-veins.

### Trichomes discovered fortuitously and may contribute to plant resistance against herbivory

Remarkably, glandular trichomes were observed on *A. ulvaceus* in the present study (Fig. 2b). No findings related to the development of trichome have thus far been reported. Located on both the adaxial and abaxial surfaces of the leaves and along the petioles, trichomes formed sparingly and were scattered over the entire surface. Of note, young leaves remained glabrous and during the third week of growth, trichome formation initiated. In the case of in vitro-raised plantlets, however, trichome development was not detected. Interestingly, a single type of detritivore was observed coexisting in the water in which the analysed plants were maintained. Therefore, we hypothesized that a resistance mechanism could have been triggered in response to damage caused by the detritivores. In an effort to test the possibility of induced trichome production, an observational analysis was conducted *ex vitro* on in vitro-raised plants whereby these were transferred to a water-filled tank containing sterile aquarium substrate. Again, trichome formation was not observed (0 out of 5). Thus, these results imply that *A. ulvaceus* is capable of induced trichome formation in response to herbivory.

### Anatomical adaptations of *A. ulvaceus* to an aquatic environment

Dissection and microscopic examination exhibited that honeycomb type aerenchyma, characterized by the irregular and large air spaces, was observed in the shoots of *A. ulvaceus* (Fig. 2c, d). Freshly cut transverse sections of leaf midrib revealed that a large proportion of the cellular volume is occupied by an air-filled lacunae system (Fig. 2d). Parenchyma cells that surround the lacunae and at least seven vascular bundles occupied the remaining cellular volume. The same midrib cross section showed the presence of small hexagonal diaphragm cells in some of the lacunae (Fig. 2e). Together, these results demonstrate the successful adaptation of *A. ulvaceus* to a submerged aquatic environment, as anatomical modifications of the shoot such as the existence of air-filled aerenchyma, the extensive lacunae system and cellular diaphragms are evident.

### Circumnutation behaviour of *A. ulvaceus* shoots

**Fig. 3:**
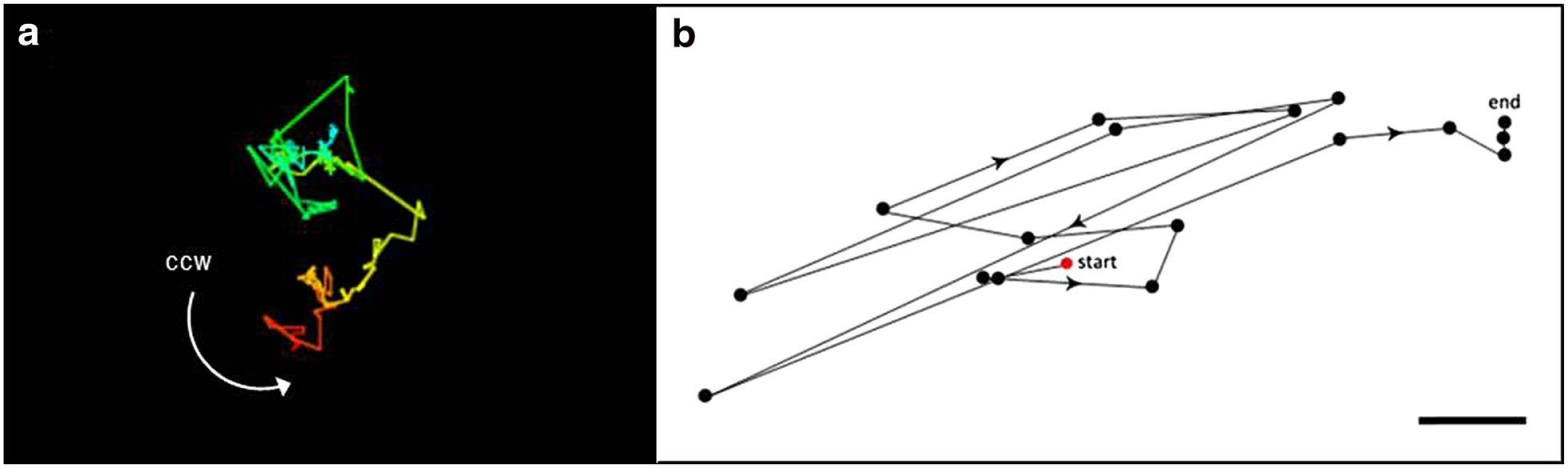
Top-view of the circumnutation trajectories of *A. ulvaceus* shoots. **a** Irregular zigzag circumnutation. Shoot movement marked by colour gradients beginning from *red* to *blue line*. Trajectory was recorded at 5 min intervals and extracted from CT. **b** Rosette-like circumnutation; *scale bar* 2 cm. *Each point* indicates tip position at 5 min intervals. Direction of movement as shown by *arrowhead*

The movements of a growing 4-day-old shoot were tracked using the time-lapse video method (Additional file 1: Video S1). Circumnutation parameters and trajectory of shoot apex were subsequently generated using *CT* software. The oscillation pattern of the shoot depicts a wide ellipse with a shape coefficient of 0.84 and it circumnutated in a counter-clockwise (ccw) direction (left-handed) (Fig. 3a). It was found that the tip organ assumes the irregularly zigzag trajectory of circumnutation, although previously it was demonstrated that rosette-like trajectory also occurs in *A. ulvaceus* (Fig. 3b). The circumnutation period of the shoot typically reaches 110 min with amplitude (length) of 77.15 mm. Taken together, these qualitative observations demonstrate the occurrence of circumnutation in the aquatic plant *A. ulvaceus*, especially in the actively growing parts of plant organs.

### In vitro regeneration of shoots and roots

**Fig. 4:**
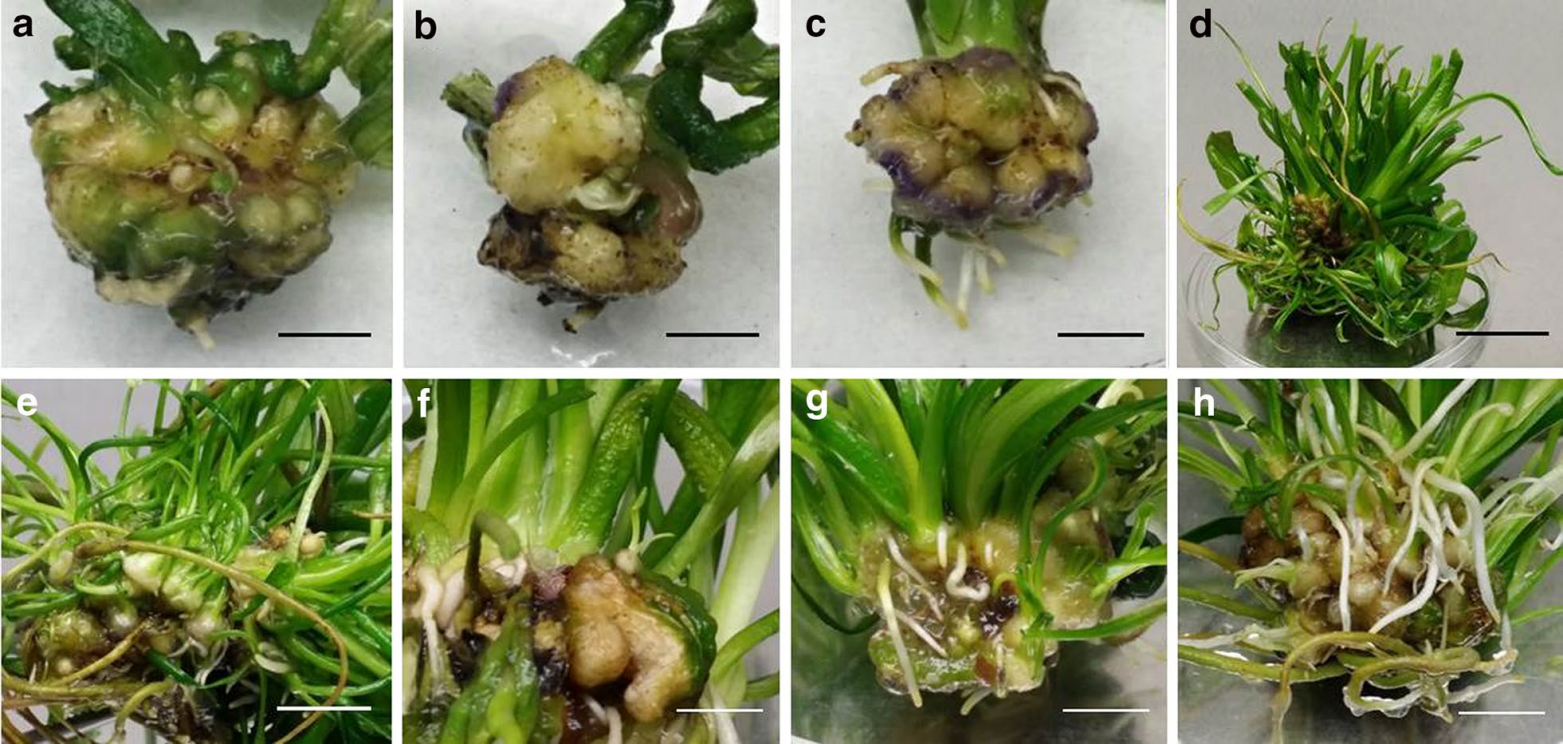
Indirect organogenesis from tuber-induced callus. **a**–**c** Developing *yellowish green* globular callus after 4 weeks. Callus formed on the edges of excised surface and proliferate on the entire surface of tubers; *scale bars* 0.5 cm. Note the *purple* colouration of callus in **c**. **d** Multiple shoot and root development from a single callus piece. Long shoots are trimmed to ease handling; *scale bar* 2 cm. **e** Multiple shoot apical meristems developing on callus; *scale bar* 0.8 cm. **f** An individual immature tuber with two shoots growing from the callus tissue; *scale bar* 0.6 cm. **g**–**h** Root development from callus tissue; *scale bars* 0.6 and 0.8 cm respectively

Indirect organogenesis of *A. ulvaceus* under the influence of different concentrations of BAP and NAA was investigated. Results on callus induction and organogenesis using immature tuber tissues with meristems are shown in Table 1. Compact, yellowish green globular calli were formed after 4 weeks of incubation (Fig. 4a–c). Among the PGR combinations tested, T5 has yielded the highest number of shoots per explant (3.67) in day 28 (Table 1). However, after 8 weeks of incubation, shoot number of T3 culture increased up to 14.7-fold as compared to 28 days of growth with an overall highest mean shoot number (50.44). It is worth noting that longer exposure time of explants to BAP and NAA is critical for shoot regeneration and subsequent mass propagation as can be seen in the significant increase in % shoots and mean number of shoots in all the treatments compared to the control (Table 1).

**Table 1:** Effects of BAP and NAA in combination on shoot regeneration from callus of *A. ulvaceus* after day 28 and 56 of culture

Treatment	Plant growth regulators (mg/L)		% shooting		Mean number of shoots per explant	
	BAP	NAA	Day 28	Day 56	Day 28	Day 56
T0	0	0	25.0^ab^	33.3^a^	0.67 ± 1.15^a^	6.92 ± 11.98^a^
T1	1	1	33.3^ab^	100.0^b^	1.75 ± 1.25^a^	22.50 ± 5.24^b^
T2	1	2	50.0^ab^	91.7^b^	2.67 ± 3.78^a^	37.17 ± 38.11^b^
T3	1	3	50.0^b^	83.3^b^	3.42 ± 1.42^a^	50.44 ± 30.63^b^
T4	2	1	25.0^ab^	100.0^b^	2.92 ± 3.17^a^	40.34 ± 12.57^b^
T5	2	2	41.7^ab^	75.0^b^	3.67 ± 1.28^a^	39.33 ± 1.91^b^
T6	2	3	66.7^b^	91.7^b^	2.17 ± 1.28^a^	31.65 ± 16.40^b^
T7	3	1	30.0^ab^	91.7^b^	0.75 ± 0.25^a^	31.33 ± 11.75^b^
T8	3	2	8.3^a^	66.7^b^	1.50 ± 2.60^a^	15.00 ± 9.69^b^
T9	3	3	50.0^b^	91.7^b^	2.25 ± 1.52^a^	29.41 ± 19.30^b^

Results represent mean ± standard deviation (SD) of three replicated experiments. Different letters in each column indicate significant difference of mean values (*P* < 0.05) using Duncan’s multiple range test

The morphogenetic response of callus to form roots on different BAP and NAA combinations is presented in Table 2. It was observed that root development commonly initiates following the development of shoots from the callus (Fig. 4d). T1 showed a similar positive effect on root regeneration that yielded an average root number of 29.50, which is significantly higher than control (i.e. 7.9-fold). The best response was observed in T4 after 56 days with the highest root number of 44.58. At 9 weeks of culture, multiple shoot apical meristems with proliferating green shoots and roots are clearly visible on the globular calli (Fig. 4e–h). Thus, these results corroborate the feasibility of *A. ulvaceus* micropropagation from callus induction using immature tuber tissues.

**Table 2:** Effects of BAP and NAA in combination on root regeneration from callus of *A. ulvaceus* after day 28 and 56 of culture

Treatment	Plant growth regulators (mg/L)		% rooting		Mean number of roots per explant	
	BAP	NAA	Day 28	Day 56	Day 28	Day 56
T0	0	0	8.3^a^	33.3^a^	0.33 ± 0.58^a^	3.75 ± 6.50^a^
T1	1	1	16.7^a^	75.0^b^	0.67 ± 0.63^ab^	29.50 ± 3.68^b^
T2	1	2	16.7^a^	58.3^b^	0.33 ± 0.38^ab^	30.00 ± 19.64^b^
T3	1	3	41.7^a^	50.0^ab^	4.25 ± 4.26^b^	31.58 ± 12.35^b^
T4	2	1	16.7^a^	91.7^b^	2.42 ± 2.27^ab^	44.58 ± 17.89^b^
T5	2	2	8.3^a^	66.7^b^	0.08 ± 0.14^a^	24.17 ± 9.15^b^
T6	2	3	8.3^a^	75.0^b^	0.42 ± 0.72^ab^	27.08 ± 7.37^b^
T7	3	1	25.0^a^	91.7^b^	1.00 ± 0.87^ab^	32.00 ± 20.22^b^
T8	3	2	8.3^a^	66.7^b^	0.75 ± 1.30^ab^	20.17 ± 8.14^b^
T9	3	3	16.7^a^	83.3^b^	1.00 ± 1.73^ab^	22.25 ± 20.88^b^

Results represent mean ± SD of three replicated experiments. Different letters in each column indicate significant differences of mean values (*P* < 0.05) using Duncan’s multiple range test

## Discussion

As aforementioned, scientific literatures of *A. ulvaceus* are lacking, especially the morphology and anatomical structures, about which little is known. Consequently, discrimination of true species from hybrids is difficult and this will affect the marketable quality of the plant. In the present study several important characteristics of this Malagasy species were identified by observational analysis. The discovery that *A. ulvaceus* is monoecious, as corroborated by the presence of both male and female reproductive organs in a single flower, is consistent with the premise that Aponogetonaceae is usually monoecious and seldom dioecious (van Bruggen 1998). Other features such as the occurrence of stamens in six and carpels in three as well as blade structures were described in details in the current study. The unique foliage shape of *A. ulvaceus*, with long, wavy and often contorted leaves indicates its adaptability to rapidly flowing water similar to other *Aponogeton* species (Hellquist and Jacobs 1998). In addition, the undulated leaf margins of *A. ulvaceus* is suggested to help minimize the boundary layer thickness in low water velocities by creating turbulence, which in turn increases the uptake of dissolved inorganic carbon (Raven et al. 1985).

In this study, we report, for the first time, the presence of glandular trichomes on leaf surfaces and petioles of *A. ulvaceus*. However, we found that none of the in vitro-cultured plants have trichomes. Likewise, leaf surfaces of in vitro-raised plants grown *ex vitro* remained glabrous. A marked difference observed was there were no detritivores (organisms feeding on dead plant materials) inhabiting the water in the *ex vitro* study, thus in vitro-raised plants were not damaged. The detritivores could have inflicted damage to live cells while feeding on dead plant parts. It is, therefore, reasonable to speculate that the leaf trichome formation in *A. ulvaceus* is a response following damage caused by herbivory. In this regard, *A. ulvaceus*, that was previously glabrous, produces new leaves with trichomes that start developing in the third week of growth which may be favourable, as it increases resistance against herbivores (Dalin et al. 2008). This is similar to the resistance mechanism of several plant species whereby new leaves with increased trichome number or density are produced (Traw and Bergelson 2003; Björkman et al. 2008). Our finding provides the first evidence that such a response occurred in *A. ulvaceus* and further field studies addressing functional and adaptive significance of induced trichome development should be evaluated in the context of effects on tritrophic interactions and plant fitness under varying abiotic conditions.

Submerged aquatic macrophytes are highly advanced as they have evolved specialized anatomical structures, enabling them to flourish in aquatic environments while growing in anoxic rooting substrate. It has been suggested that aerenchyma is responsible for the oxygen supply to aquatic plant roots, thereby facilitating internal oxygen transport and enhancing metabolic efficiency (Armstrong et al. 1991). The aerenchyma lacunae form a relatively continuous pipeline system through plants which generally provide a pathway that allows adequate aeration of roots, supporting respiration of submerged tissues (Maricle and Lee 2002). However, Rascio (2002) pointed out that while emergent and floating-leaf plants are dependent on the internal oxygen transport from leaves to roots via the air lacunae system, submerged species acquire these gases through diffusion. Thus, leaves of the latter species are usually thin, translucent and do not possess developed aerenchyma, if leaves are entire. Interestingly, in *A. ulvaceus*, the presence of honeycomb-like aerenchyma and an extensive lacunae system were observed in the midrib. On the basis of such evidence, *A. ulvaceus* plants could have evolved to adapt in both lotic and lentic environments. It is possible that during seasonal dry periods, leaves are exposed to the oxygen-rich atmosphere, thus *A. ulvaceus* will rely on the aerenchyma for oxygen supply since the diffusion coefficient of oxygen transport in water is much lower than in air (Aachib et al. 2004). Similarly, respiration of *A. ulvaceus* when fully submerged underwater will be facilitated by diffusion via its leaves, as oxygenation is higher due to turbulence in the lotic environment (Lusa et al. 2011). The existence of cellular diaphragms within the lacunae, exhibiting a marked cell dimorphism, is yet another anatomical adaptive strategy found in *A. ulvacues*. Apart from providing lateral support to stem and cross bundles, other functions of diaphragms include tannin storage (Snow 1914), carrying laticifers (Stant 1964), photosynthetic capability (Kaul 1973) and preventing internal flooding caused by injury (Soukup et al. 2000).

The twisting and rotating movements of rapidly elongating plant organs (hypocotyl, tendril, shoot or root) have long became a fascination to biologists and have spawned a variety of studies on terrestrial plants. In the present investigation, the occurrence of circumnutation has been demonstrated in the aquatic plant *A. ulvaceus* using the novel software *CT*. The mechanisms and functional importance underlying circumnutation has long been debated since Darwin’s time. At present, it is presumed that circumnutation is dependent upon the internal oscillatory movement and gravity sensing. In the hydrophyte *Vallisneria*, however, female flowers are capable of circumnutating in the absence of gravitropism. The paper by Kosuge et al. (2013) indicated that the helical intercalary growth of the peduncle, the internal oscillator, is the driving force of circumnutation. As buoyancy minimizes the gravity effect, which renders it difficult to study circumnutation in land plants due to the innate nature of gravitropic response, shoot movements underwater are interesting. Then again, mutant analyses on agravitropic mutants of morning glory that lack starch-filled amyloplasts in endodermal cells were defective in shoot circumnutation, indicating the importance of gravitropic response (Kitazawa et al. 2005). Additionally, there is increasing evidence that circumnutations possess distinct ecological functions and are not simply a way of growth. In *Oryza sativa*, circumnutating roots have a significant role in seedling establishment on waterlogged and soft soil (Inoue et al. 1999). Likewise, circumnutation in *Vallisneria* is crucial for hydrophilous pollination on the water surface (Kosuge et al. 2013). In this regard, circumnutation proves to be a complex phenomenon and dependent on plant species, the organs involved and the developmental stage (Kitazawa et al. 2005; Stolarz 2009). Clearly, further analysis is warranted to elucidate the molecular mechanism of circumnutation in *A. ulvaceus* as well as the implicated ecological functions of shoot movements.

The procedure described here is the first successful in vitro micropropagation system for *A. ulvaceus* via indirect organogenesis. Based on the evidence, immature tubers with meristems were the most successful explant in callus induction and subsequent organogenesis. Due to their physiological state of being young, healthy and nutrient-rich, immature tubers are ideal explant sources. Younger explants exhibiting greater morphogenic potential has already been established (Yepes and Aldwinekle 1994) owing to their meristematic properties and increased totipotency in vitro (Razdan 2003). However, in other sources such as leaves, petioles and root tips of *A. ulvaceus*, lethal browning of tissue was one of the impediments to callus induction. Our findings are in conformity with the results of Carter and Gunawardena (2011), who observed no callus formation in petioles and root tips of *A. madagascariens* due to browning. Such phenomenon can be attributed to the exudation and oxidation of phenolic compounds in the culture medium as a defence response following tissue wounding or stress (Jones and Saxena 2013).

In *A. ulvaceus*, the BAP and NAA combination was found to be suitable for callus induction and organogenesis. This hormonal combination has been extensively utilized for indirect organogenesis in various protocols developed for other plant species of *Aponogeton* (Carter and Gunawardena 2011), *Artemisia* (Tariq et al. 2014) and *Saussurea* (Zhao et al. 2001; Dhar and Joshi 2005). Taking into consideration that indirect morphogenic routes can generate somaclonal variation in micropropagated progenies causing both genetic and epigenetic alterations (Ramírez-Mosqueda and Iglesias-Andreu 2015), these genetic variabilities can be exploited for genetic improvement of ornamental species with growing economic importance such as *A. ulvaceus* (Sun et al. 2013; Krishna et al. 2016).

In the present investigation, regeneration capacity of *A. ulvaceus* shoots and roots was significantly enhanced with the addition of BAP and NAA to MS medium. Maximum root numbers (45 roots per explant) were produced in response to 2 mg/L BAP and 1 mg/L NAA (T4). These results are in agreement with Oh et al. (2008), who reported that a lower concentration of auxin is optimal for rooting. Under the same treatment condition, however, shoot proliferation response was onefold (40 shoots per explant) lower than that obtained with 1 mg/L BAP and 3 mg/L NAA (T3) (50 shoots per explant). This suggests the importance of the ratio of auxin (NAA) to cytokinin (BAP) in the manipulation of plant regeneration in *A. ulvaceus*. The significance of the auxin to cytokinin ratio in culture medium has been highlighted by several reports. It was indicated that auxins at lower concentrations along with cytokinins are critically important in plant regeneration in a number of systems such as *Coffea arabica* (Zoriniants et al. 2003), *Eleusine indica* (Yemets et al. 2003) *Juncus effusus* (Xu et al. 2009) and *Senecio candicans* (Hariprasath et al. 2015). Though findings in relation to the effectiveness of culture media supplemented with BAP (2 mg/L) and NAA (1 mg/L) on shoot regeneration have been reported in *S. candicans* (Hariprasath et al. 2015), the differential response may be attributed to the inhibitory effect of higher BAP concentrations on shoot multiplication. Similar observations on the inhibitory effect of BAP were also reported in *Tinospora cordifolia* (Raghu et al. 2006) and *Sida cordifolia* (Sivanesan and Jeong 2007).

With no statistical difference between PGR treatments on shoot regeneration, the best responses were found in MS media supplemented with 1 mg/L BAP and 3 mg/L NAA, and 2 mg/L BAP and 1 mg/L NAA. However, in the latter treatment, it was shown to be more effective on root regeneration. Despite the increased regeneration capacity induced by both treatments, it was found that suboptimal shoot and root numbers of 22.50 and 29.50, respectively, were significantly produced with 1 mg/L BAP and 1 mg/L NAA (T1) when compared to control explants. Based on these observations, it appears that *A. ulvaceus* is rather responsive to phytohormones, as the addition of low concentrations of PGR is sufficient to induce regenerative responses in tuber explants. Our results are in agreement with recent reports on *Humulus lupulus* whereby explants exhibited high sensitivity to changes in auxin concentration and low IAA concentration was beneficial for shoot initiation from callus tissues (Trojak-Goluch et al. 2015). As highlighted by Hill and Schaller (2013), plants capable of accumulating higher levels of bioactive cytokinin are generally more receptive to methods employed for the induction of *de novo* shoot organogenesis and in vitro plant regeneration. It is possible that the presence of the mechanisms necessary for plant regeneration may positively impact *A. ulvaceus* regenerative capability, allowing it to readily undergo callus induction and organogenesis. Another possible explanation is that tuber or bulb explants having larger nutrient reserves tend to readily regenerate in vitro and are more independent of the hormones in growth medium (Yildiz 2012).

In consideration of the production cost of in vitro mass propagation of *A. ulvaceus*, the use of lower concentrations of plant hormones is more cost effective. Also, low hormone concentrations are often associated with lower incidence of or no somaclonal variation (Swartz 1991). Thus, it can be concluded that MS medium containing 1 mg/L BAP with 1 mg/L NAA was the optimal combination for callus induction and subsequent organogenesis of *A. ulvaceus*. This protocol could potentially be used for the commercial propagation of this economically valuable aquatic plant. In addition, with the present regeneration system, further research should focus on evaluating: (1) the functional and adaptive significance of induced trichome formation; (2) the underlying molecular mechanism of circumnutation in *A. ulvaceus* and its implicated ecological functions of shoot movements; and (3) the mutational possibilities during regeneration which could affect plant behaviour including circumnutation.

## Conclusion

This study provides useful information on the biology of the ornamental plant *A. ulvaceus*. Through close examination of *A. ulvaceus*, glandular trichomes were found to be present on the leaves. By recording time-lapse videos to track the movement of shoots, it was found that the shoots moved in a specific trajectory known as circumnutation. The study on the morphology, anatomy and physiology of *A. ulvaceus* will provide a more in depth understanding of the plant as well as its functions and applications.

Since *A. ulvaceus* is a much sought after ornamental aquatic plant, mass multiplication of the plants is essential for preserving this rare species in their natural habitat. We have successfully developed an in vitro regeneration protocol in this study through an intermediate callus phase using MS medium supplemented with 1 mg/L BAP and 1 mg/L NAA. This protocol will provide an alternative method with the potential to mass propagate *A. ulvaceus*.

## Additional file

10.1186/s40064-016-3041-4 Time-lapse video of shoot circumnutation in *Aponogeton ulvaceus*.
